# Supplementary material for: MetaRibo-Seq measures translation in microbiomes
Source: Nat Commun. 2020 Jun 29;11:3268. doi: 10.1038/s41467-020-17081-z (PMC7324362; doi:10.1038/s41467-020-17081-z)
Supplement: Supplementary file 10 — Supplementary Data 7 [file 41467_2020_17081_MOESM10_ESM.zip › File2/Confidence_High_Taxonomy/322739_out.krona.html]

Javascript must be enabled to view this page.

members
magnitude
magnitudeUnassigned
count
unassigned
taxon
rank

322739\_out

5

5
2
superkingdom

1239
5
phylum

186801
5
class

order
186802
5

1
31979
family

1
1485
genus

species

SRS015960\_contig\_number\_16814
1
59620

family
1
186806

1
1730
genus

species
1

SRS017307\_contig\_number\_33017
142586

family
186803
3

189330
1
genus

species

SRS064557\_contig\_number\_6723
1
286138

genus
2
33042

species

SRS050422\_contig\_number\_8617SRS1041091\_contig\_number\_17202
2
410072
